# The Normalization of Vaping on TikTok Using Computer Vision, Natural Language Processing, and Qualitative Thematic Analysis: Mixed Methods Study

**DOI:** 10.2196/55591

**Published:** 2024-09-11

**Authors:** Sungwon Jung, Dhiraj Murthy, Bara S Bateineh, Alexandra Loukas, Anna V Wilkinson

**Affiliations:** 1 School of Journalism and Media University of Texas at Austin Austin, TX United States; 2 University of Texas Health Science Center at Houston School of Public Health Houston, TX United States; 3 Department of Kinesiology and Health Education University of Texas at Austin Austin, TX United States

**Keywords:** electronic cigarettes, vaping, social media, natural language processing, computer vision

## Abstract

**Background:**

Social media posts that portray vaping in positive social contexts shape people’s perceptions and serve to normalize vaping. Despite restrictions on depicting or promoting controlled substances, vape-related content is easily accessible on TikTok. There is a need to understand strategies used in promoting vaping on TikTok, especially among susceptible youth audiences.

**Objective:**

This study seeks to comprehensively describe direct (ie, explicit promotional efforts) and indirect (ie, subtler strategies) themes promoting vaping on TikTok using a mixture of computational and qualitative thematic analyses of social media posts. In addition, we aim to describe how these themes might play a role in normalizing vaping behavior on TikTok for youth audiences, thereby informing public health communication and regulatory policies regarding vaping endorsements on TikTok.

**Methods:**

We collected 14,002 unique TikTok posts using 50 vape-related hashtags (eg, *#vapetok* and *#boxmod*). Using the k-means unsupervised machine learning algorithm, we identified clusters and then categorized posts qualitatively based on themes. Next, we organized all videos from the posts thematically and extracted the visual features of each theme using 3 machine learning–based model architectures: residual network (ResNet) with 50 layers (ResNet50), Visual Geometry Group model with 16 layers, and vision transformer. We chose the best-performing model, ResNet50, to thoroughly analyze the image clustering output. To assess clustering accuracy, we examined 4.01% (441/10,990) of the samples from each video cluster. Finally, we randomly selected 50 videos (5% of the total videos) from each theme, which were qualitatively coded and compared with the machine-derived classification for validation.

**Results:**

We successfully identified 5 major themes from the TikTok posts. *Vape product marketing* (1160/10,990, 8.28%) reflected direct marketing, while the other 4 themes reflected indirect marketing: *TikTok influencer* (3775/14,002, 26.96%), *general vape* (2741/14,002, 19.58%), *vape brands* (2042/14,002, 14.58%), and *vaping cessation* (1272/14,002, 9.08%). The ResNet50 model successfully classified clusters based on image features, achieving an average *F*_1_-score of 0.97, the highest among the 3 models. Qualitative content analyses indicated that vaping was depicted as a normal, routine part of daily life, with TikTok influencers subtly incorporating vaping into popular culture (eg, gaming, skateboarding, and tattooing) and social practices (eg, shopping sprees, driving, and grocery shopping).

**Conclusions:**

The results from both computational and qualitative analyses of text and visual data reveal that vaping is normalized on TikTok. Our identified themes underscore how everyday conversations, promotional content, and the influence of popular figures collectively contribute to depicting vaping as a normal and accepted aspect of daily life on TikTok. Our study provides valuable insights for regulatory policies and public health initiatives aimed at tackling the normalization of vaping on social media platforms.

## Introduction

### Background

TikTok is a short video–sharing platform that has grown in popularity, primarily among youth and young adults, since its launch in 2016. Although TikTok restricts uploading videos containing “the depiction, promotion, or trade of drugs or other controlled substances,” such content persists on the platform. The e-cigarette or vaping industry and provaping influencers are increasingly using social media platforms, including TikTok, to disseminate content that glamorizes and normalizes vaping [1,2]. This surge of promotional content is concerning because it shapes perceptions, especially those of younger people, suggesting that vaping is a desirable and socially acceptable behavior [3]. By depicting vaping in a humorous light and portraying it as cool, as well as associating it with a particular lifestyle, such content can indirectly send misleading messages, implying that vaping is an integral and normal aspect of daily life [4]. In addition, despite existing policies, some e-cigarette retailers continue to directly market e-cigarette products on social media in ways that are explicitly designed to appeal to specific targeted audiences, such as youth [5]. This includes offering exclusive deals, using attractive packaging, and introducing a variety of flavors. The underlying strategies of these marketing efforts are to desensitize people to the presence and use of e-cigarettes [6], making it seem that vaping is a routine part of everyday life [4]. This trend is alarming because it downplays the health risks associated with vaping and shapes the attitudes and behaviors of young people who are vulnerable and open to suggestion [7].

Previous work based on qualitative content analysis has examined how TikTok posts depict e-cigarettes and vaping [1,2]. These studies are based on manual, human coding, which requires a substantial number of labor hours, a method that does not effectively scale to the large volume of content currently produced and consumed on TikTok [8,9]. This study uses a hybrid method to analyze a large set of TikTok posts depicting e-cigarettes and vaping. A hybrid method combines computational and qualitative thematic analysis of textual and visual data, respectively, to ensure precision and reduce bias. This approach extends existing research by examining both linguistic (ie, text) and visual (ie, video) elements of e-cigarette content on TikTok. The purpose of this study is to identify direct or indirect themes promoting e-cigarettes on TikTok and to describe how these themes might play a role in normalizing vaping behavior on TikTok for youth audiences.

### The Normalization of Vaping

Normalization in sociology refers to the process by which an individual adjusts their behavior, thoughts, beliefs, or feelings to conform to the social norms and expectations of their culture or group [10]. This process can occur through repetition, ideology, and propaganda [11]. The normalization of vaping refers to the process by which vaping becomes perceived as a typical, socially acceptable behavior within society [12,13]. The shift in perception and attitude typically occurs gradually through multiple factors that interact and reinforce each other, such as widespread advertising, media representation, cultural trends, peer influences, product accessibility, and perceived safety and harm reduction messages [14-17].

The marketing of vaping devices and flavor options across digital platforms and print media could play an important role in shaping public perceptions of vaping [14]. Similarly, the portrayal of vaping in movies, television shows, and web-based content contributes significantly to its normalization, especially when famous characters and models are depicted using e-cigarettes, subtly implying that vaping is acceptable and trendy [15]. Peer influence also has a strong impact on individuals’ attitudes toward vaping; if vaping is common within a peer group, others in the group are more likely to vape to fit in [16]. The ease of accessing e-cigarettes, boosted by web-based sales and in the absence of strict regulations, further increases their use. Finally, the perception of vaping as a safer alternative to traditional smoking, driven by harm reduction messages, lowers barriers to experimentation and regular use [18]. All these factors collectively create an environment where vaping is increasingly normalized, especially among younger people.

The normalization of vaping carries considerable implications for public health, especially if the risks associated with vaping trends are not adequately addressed or recognized; for instance, research shows that the normalization of vaping, especially among youth, can increase dependence risk, normalize smoking behavior, and potentially lead to the initiation of traditional tobacco cigarettes, especially among those who might never have smoked otherwise [19,20].

### Social Media Marketing Strategies and the Normalization of Vaping

Social media platforms have become a pivotal tool in shaping public behaviors in recent years [21,22]. TikTok, for instance, offers users the opportunity to share their thoughts, experiences, and preferences to a vast audience [23]. Recent data indicate a surge in content related to vaping use on these platforms. Notably, the majority of messages are positive and favorable toward vaping [24]. Content in these messages ranges from personal testimonials about the benefits of vaping over traditional smoking to endorsements by influencers of specific e-cigarette brands and flavors [25]. Such messages might lead to gradually establishing the belief that vaping is a normalized and socially acceptable behavior [26,27], regardless of any associated potential health risks or controversies [28,29].

Both direct (ie, explicit promotional efforts of e-cigarettes) and indirect (ie, subtler strategies presenting the promotion in a lifestyle-oriented manner) marketing through social media platforms, including TikTok, significantly influence the popularity of e-cigarette use [1,2]. Individuals who are exposed to promotional or user-generated e-cigarette content have a lower perception of harm, have more positive attitudes toward vaping, and have a higher risk of initiating e-cigarette use [26,27]; for example, a recent longitudinal study found that adolescents who reported seeing e-cigarette posts at least 1 time a week on TikTok had a higher risk of lifetime and current e-cigarette use [24]. The findings also indicated that the frequency of TikTok use was a significant predictor of initiating e-cigarette use [24]. The findings from another study indicated that when young people on social media (friends, influencers, etc) are portrayed as happy and popular while using e-cigarettes, it creates a perception that vaping is a normative behavior within their social circles and, as such, is a behavior to emulate [30].

TikTok’s community guidelines [31] regarding “drugs, controlled substances, alcohol, and tobacco” clearly state that users should not share content that involves “the purchase, sale, trade, or solicitation of drugs or other controlled substances, alcohol or tobacco products (including e-cigarettes).” However, e-cigarette content is prevalent on TikTok, often masked by indirect advertising methods [32]. Instead of traditional advertisements, e-cigarette brands collaborate with influencers to promote their products in their daily life posts and videos, making the promotion seem more organic. Influencers also link e-cigarette use with appealing lifestyles, such as wellness, party scenes, or luxury [2]. The repetitive e-cigarette content posted by influencers could potentially normalize the act of vaping and make users more receptive to vaping, especially if the content is presented without highlighting potential risks. This psychological phenomenon, often termed the “mere exposure effect,” suggests that people tend to develop a preference for behaviors merely because they are familiar with them [33].

In addition, content featuring individuals from different backgrounds engaging with e-cigarettes has the potential to appeal to wider demographics. Individuals often connect more with content that mirrors their own experiences, identity, ethnicity, and age, which may prompt them to think about experimenting with e-cigarettes [34]. The dynamic interplay and use of these indirect marketing strategies on TikTok and other platforms contribute to the normalization of vaping by integrating it into popular culture, social practices, and lifestyle images among various backgrounds*.* Thus, in this study, we aimed to identify and describe themes—either direct or indirect—that capture how e-cigarettes are promoted on TikTok and understand how these themes might play a role in the perception and normalization of vaping among young people.

### The Need to Use Computational Methods to Study Vaping on Social Media

Given the rising popularity of TikTok as a social media platform, several qualitative studies have successfully examined e-cigarette–related content on TikTok. Sun et al [30] found that the themes capturing e-cigarettes depicted in TikTok videos included humor and jokes, lifestyle, marketing, vaping tricks, nicotine and addiction, creativity, and caution. Purushothaman et al [35] concluded that more than two-thirds of the posts contained references to e-cigarettes. Although these studies advance our understanding of how e-cigarettes are depicted on social media, they are limited to the examination of a small number of posts because the content was analyzed manually. The volume of social media posts has rapidly risen and outpaced thematic analysis based on manual content analysis. Computational methods—both natural language processing and computer vision—can help researchers analyze e-cigarette–related content on social media at scale [36,37].

Media scholars increasingly use computational analytic methods (eg, developing a computational interface to facilitate the work of human coders) to manage ever-larger data sets [38]. Computational analytic methods have the potential to overcome many of the sampling and coding limitations of conventional content analysis. Early studies in computational social science focused on the TikTok platform have primarily concentrated on text analysis using natural language processing [39]. In the vast domain of health care, natural language processing serves as a powerful tool with diverse applications, offering insights and solutions to various challenges faced by clinicians, researchers, and health care providers [40]. Natural language processing techniques are instrumental in mining textual data from web-based health forums, social media platforms, and patient-generated content [41]. By analyzing conversations, posts, and comments, natural language processing algorithms can identify emerging health trends, public perceptions of diseases and treatments, adverse drug reactions, and even sentiments toward health care providers and services; for example, Sun et al [42] used topic modeling, a form of natural language processing and machine learning that automatically identifies topics or themes in a collection of documents [8], to examine COVID-19 vaccine–related posts on TikTok through textual data. Furthermore, using natural language processing, Bharti et al [43] created a multilingual conversational bot to deliver essential health care education, information, and advice to individuals with chronic conditions.

More recent social media research has extended the scope of computational analytic methods to encompass computer vision techniques, a method designed for analyzing social media images and videos [44,45]. Computational techniques rooted in deep learning have facilitated the automated examination of vaping-related visual content shared on social media platforms such as Instagram and TikTok [45]. Moreover, machine learning models based on computer vision have been found to effectively and precisely recognize e-cigarette–related content (eg, vaping devices and vapor clouds) on predominantly visually oriented social media platforms [44]. The transition from text-focused analysis to computer vision analysis signifies an evolution in TikTok research methodologies, offering a more comprehensive understanding of the platform’s multifaceted content and societal implications. Nevertheless, there is still a dearth of studies applying computational content analysis methods to the context of vaping. Research is particularly lacking in the realm of combining computational methods with both text and images, especially to detect vaping-related themes.

### The Fundamentals of Computational Image Clustering

Computational image clustering is a popular computer vision method that groups images based on visual similarity [46]. Computer vision involves developing algorithms and models to help computers interpret and understand images and videos [47]. To mimic human vision, computer vision teaches machines to recognize objects, scenes, and patterns as well as understand visual inputs [48]. Computer vision performs object recognition and content categorization using image features, patterns, and structures [49]. The goal is to find patterns or similarities in a large number of images without knowing their content or categories. Each cluster comprises images with similar visuals. This procedure enhances visual data navigation and comprehension, particularly for large image collections. There are 2 steps to image clustering: feature extraction and feature-based clustering.

Several machine learning computational models are capable of performing feature extraction. In this step, visual attributes from the input images are extracted. Initially, visual elements such as edges, patterns, and colors may be included [46]. The ultimate goal is to identify objects or patterns in images and capture more complex and higher-level features. Image pixel data are converted into numerical representations for computational analysis in this process [50]. Clustering algorithms group similar images into coherent categories after extracting these visual features [51]. The algorithms categorize images into meaningful groups to facilitate retrieval, analysis, and interpretation.

A convolutional neural network (CNN) can extract features and cluster images. CNNs are artificial intelligence models that specialize in image classification [52]. The main steps in the CNN procedure are feature extraction and classification [53]. Vision transformers (ViTs) are a deep learning model architecture commonly used in computer vision tasks. Transformers are a deep learning model architecture designed for natural language processing tasks, characterized by their attention mechanism and ability to capture long-range dependencies [54]. Transformers’ self-attention and parallelization make them good at sequential data processing [55]. K-means clustering is a popular unsupervised machine learning method for splitting a data set into nonoverlapping clusters. This algorithm determines data similarity [56]. Using the feature vectors from the previous step, k-means clustering groups similar images [57].

### Using Computational Image Clustering on Vape-Related Content on Social Media

Multimodal content clustering has not been used to study the normalization of vaping on social media. However, image clustering using computational methods (ie, CNNs and k-means clustering together) has been used successfully to study visual content on social media; for example, Ketonen and Malik [58] used residual network (ResNet) and Visual Geometry Group CNN models with k-means clustering to analyze and describe Instagram posts related to vaping. The authors found 7 clusters—*e-liquid*, *e-liquids*, *e-cigarettes*, *product packages*, *people*, *statements*, and *miscellaneous*—with an accuracy of 90.76%. One limitation of the study was that it relied solely on posts under a single hashtag, which raises the possibility that not all posts were directly relevant to e-cigarettes [58]. Furthermore, the use of images from the identified clusters for training a classifier for predicting a category for each image in the data set may result in the inclusion of certain inaccuracies within the final group labels. Vassey et al [59] measured the influence of e-cigarettes on Instagram using the Inception v3 model, one of the CNN-based models developed by Google, which correctly classified >90% of the images containing men, women, e-juices (the liquid used in e-cigarettes), and mod system devices (a type of e-cigarette that allows users to customize their vaping experience) across all samples. However, the Inception v3 model’s training was limited to recognizing a single class per image although specific images contained 2 distinct categories.

Setiawan and Purnama [60] applied the DarkNet19 CNN architecture to extract image features from tobacco leaf images. The authors used k-means to cluster these images (with an accuracy ranging from 0.81 to 0.93 for all 3 classes). Previous research has successfully integrated feature extraction techniques using CNN architectures based on machine learning and clustering methods. However, previous research was restricted to the use of posts under a single hashtag, and the model used needed to be updated for strong, robust performance to overcome the limitations of previous social media studies related to vaping by collecting large amounts of data using multiple hashtags and using a model with robust performance.

This study aimed to use a hybrid computational and qualitative approach to analyze a substantial volume of TikTok content related to e-cigarettes, using k-means clustering and pretrained ViT and CNN models. By identifying direct and indirect themes in the promotion of e-cigarettes on TikTok, we sought to elucidate how these themes contribute to the normalization of vaping behavior among youth audiences. This comprehensive analysis addresses gaps in existing research methodologies and provides insights into the multifaceted factors influencing the portrayal and perception of vaping on social media platforms. The findings from this study are necessary to inform the development of interventions targeting adolescents and young adults that counteract the glamorization of vaping, as evidenced by the strategies used by e-cigarette companies [61]. The findings may also be used to inform legislative actions, educational campaigns, and digital interventions aimed at denormalizing harmful behaviors and safeguarding public health, a need further emphasized by recent findings on the prevalence of provaping content on platforms such as TikTok [62].

## Methods

### Hybrid Approach

**Figure 1:**
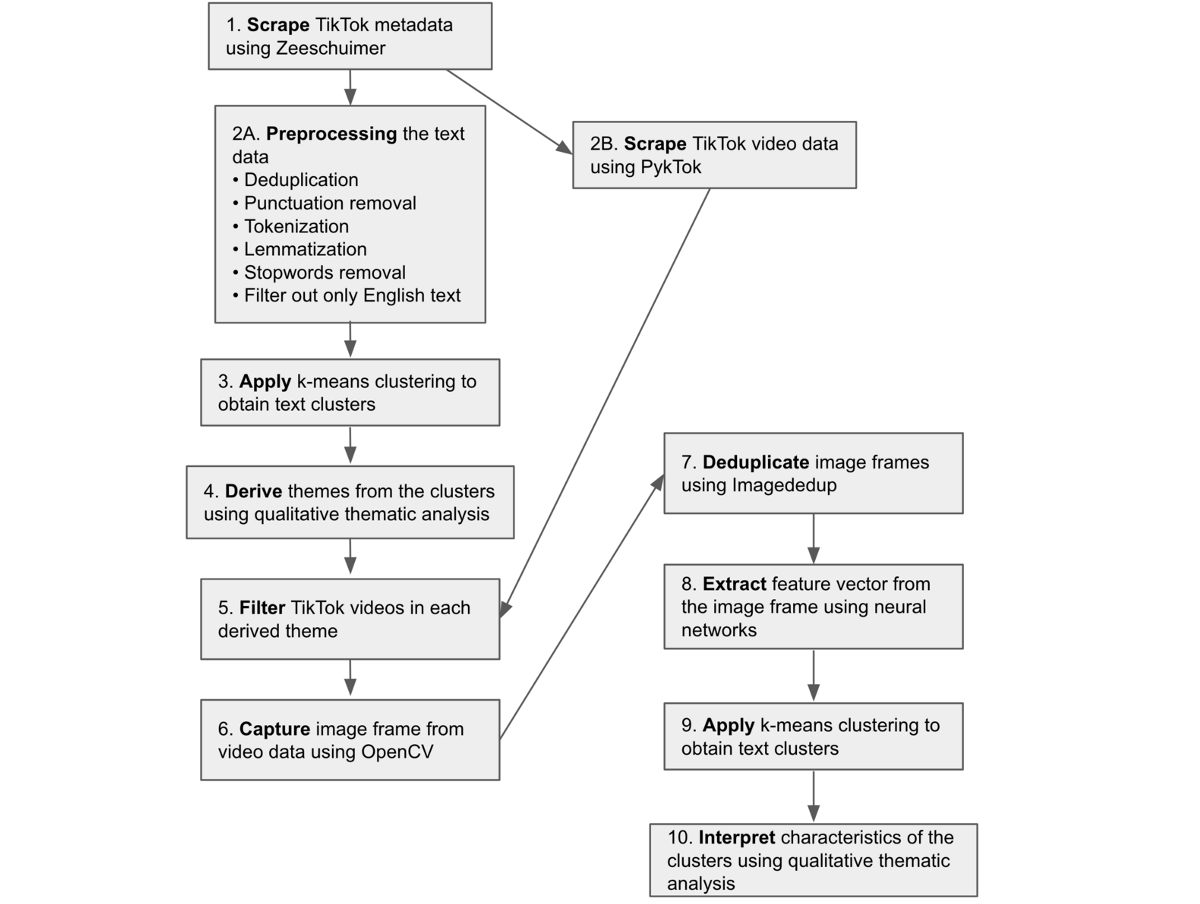
Project architecture (relevant figures and tables are referenced in parentheses). OpenCV: Open-Source Computer Vision Library. TF-IDF: term frequency–inverse document frequency.

We used a hybrid approach—computational as well as qualitative methods—to derive vaping-related themes from TikTok posts (Figure 1).

We collected 18,946 TikTok posts published from 2018 to 2023 using a set of vape-related hashtags (eg, *#vapetok*, *#boxmod*, and *#vaper*; refer to Multimedia Appendix 1 for the complete list of hashtags). Although TikTok has a research application programming interface (API) for academics, it often returns inaccurate metadata; therefore, we used a data scraping approach, which obtained accurate data and metadata. Data scraping was performed in 2 steps. First, we used Zeeschuimer [63], a Firefox browser extension that facilitates the scraping of data from social media platforms, including Twitter, Instagram, TikTok, and LinkedIn. Prior studies have demonstrated the efficacy of Zeeschuimer as a data mining tool [64,65]. In accordance with previous studies, we used the Zeeschuimer tool to obtain e-cigarette–related content from TikTok’s social media data.

**Figure 2:**
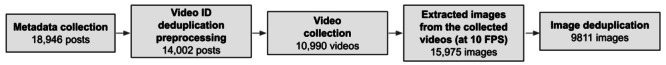
Primary data set evolution for the analysis: a flowchart of the data processing pipeline, starting with metadata collection, followed by video ID deduplication, extraction of images, and finally image deduplication. FPS: frames per second.

Zeeschuimer enables the export of the collected metadata in newline delimited JSON format. To scrape the data using Zeeschuimer, we scrolled down the TikTok hashtag URLs for each targeted hashtag. We collected 20,575 newline delimited JSON files for each hashtag. The metadata fields included fundamental video information such as *video_id*, *video_timestamp*, *video_duration*, *video_description*, *video_is_ad*, *video_height*, *video_duration*, *video_location*, and *author_name*, as well as user engagement elements such as *video_sharecount*, *video_commentcount*, and *video_playcount*. Next, we augmented our data set with an additional step: we collected TikTok videos using the *Pyktok* [66] package. *Pyktok* obtains data directly from the JSON objects embedded within TikTok web pages and from APIs that are not publicly documented. On the basis of the *video_id* collected using Zeeschuimer, we verified the valid *video_id* that is public and downloaded the TikTok videos. Accordingly, we obtained 18,946 data points for 50 hashtags (Figure 2).

### Ethical Considerations

This study underwent review by the institutional review board at the University of Texas at Austin (STUDY00006075) and was determined not to constitute human participant research, as defined by regulations set forth by the Department of Health and Human Services and the Food and Drug Administration. Consequently, institutional review board review and approval were not required for this study.

As the data used in this study are publicly available, informed consent was not required. To ensure confidentiality and privacy, all data were anonymized. Specifically, we took measures to prevent the identification of the individuals whose posts were used for analysis, including blurring identifiable information such as user IDs or TikTok IDs in any images used.

### Text Clustering Methods

We combined the textual data from the *video_description* column. We then preprocessed the data using the Python *NLTK* library [67] to perform lemmatization (finding the basic form of the word inflected in multiple forms in a sentence), punctuation removal, and capitalization removal. All collected text was tokenized or divided into word tokens, which are grammatically indivisible language elements. After the basic text preprocessing, we used the Python package *langdetect* [68] to detect and filter out only English text. Subsequently, we deduplicated the data set, which resulted in 14,002 posts, to define the clusters. As the TikTok data set lacked correct answer labels, we created a cluster using the k-means algorithm, an unsupervised learning method. The document was first converted to a vector using the term frequency–inverse document frequency (TF-IDF) method. The TF-IDF weight is a statistical value used in information retrieval and text mining that indicates how important a word is in a specific document when there is a document group consisting of many documents [69]. We set the minimum document frequency to 5 as a parameter of the TF-IDF vectorizer. Words that appeared fewer than 5 times were excluded from the word dictionary. In addition, we set the *ngram_range* to (1, 2), which means that up to 2 groups of words were treated as if they were 1 word.

Next, we normalized the word score of each document vector to keep it between 0 and 1 so that vector clustering could be performed effectively. We used cosine similarity between vector distances to perform clustering. We used k-means clustering as a computational clustering method. This is a clustering method in which the sum of the squared distances between the average vector in each cluster and the vectors in the cluster is minimized. As the center point is chosen at random, the clustering result may be different or poor. To determine the optimal number of clusters (k), we used the elbow method, which has been proven to be an excellent technique [70]. The elbow method is selected after considering the section where the inertia value, representing the sum of the distances between clusters, drops rapidly as the optimal k.

After identifying the optimal number of text clusters, we conducted qualitative textual thematic analysis to assess the coherence and relevance of the clusters, further validating the robustness of our findings. We met twice as a group to evaluate the uniqueness of the themes derived from the elbow method, and the final themes were decided by consensus. We investigated the posts that belonged to each cluster in depth, analyzed the data composition of each cluster, and grouped the posts into themes.

### Video Clustering Methods

After conducting the thematic analysis on the text corpus, we conducted image clustering on the derived themes. We examined whether the themes could be categorized according to the videos’ visual elements. Within the themes, we also identified subclusters. The average length of the videos with themes was between 22 and 31 seconds. Theme 1 encompassed 2741 videos, theme 2 included 1272 videos, theme 3 had 1160 videos, theme 4 had the largest collection of 3775 videos, and theme 5 included 2042 videos.

We initially sampled every 25th video from the total data set. We discovered that if the video lasted longer than 10 seconds, most of the first few seconds were not devoted to vaping. Following this observation, to capture the vape-related moments in the TikTok posts, we chose a frame rate of 10 frames per second and extracted frames at regular intervals of 10 frames per second from each collected video. This approach was used to mitigate any potential bias that may arise from the researcher’s subjective selection process.

Subsequently, the Python-based *Imagededup* [71] package was used to eliminate duplicate images. As mentioned previously, the *Imagededup* package has been widely used in computer vision research, demonstrating its efficacy in image deduplication [72,73]. The software package used a built-in CNN encoder for image deduplication, and the CNN encoder used the *mobilenet_v3_small* model to extract features from the images in the original image directory.

By computing the cosine similarities after the encoding of the images, the CNN algorithm selectively identified and removed only those image files duplicated from the original image directory. Cosine similarity is a mathematical metric used to compare the similarity of 2 vectors, extended to image comparison [74]. To preserve visual features, the CNN algorithm encoded each image into a vector. When these vectors were compared using the cosine similarity metric, the outcome indicated how similar the 2 images were regarding visual content. A cosine similarity score of 1 indicated that the images were identical, whereas a score of 0 indicated dissimilarity. The strict cosine similarity threshold score was set at 0.85, which means that only image pairs with a cosine similarity score of ≥0.85 would be recognized as duplicates. Images with a lower similarity score would be considered distinct.

After deduplication, we obtained 2520 (65.74%) images for theme 1 from 3833 images. The same procedure was replicated for themes 2, 3, 4, and 5. We obtained 1242 (57.18%) images after removing duplicates from a pool of 2172 images for theme 2. In addition, we obtained 1269 (69.69%) images for theme 3 from 1821 images, and 4276 (57.05%) images for theme 4 from 7495 images. Finally, we obtained 1773 (71.64%) images for theme 5 from 2475 images.

### CNN-Based Clustering

The CNN model was trained on a large data set to learn visual features, which is referred to as pretraining [75]. To classify images, feature extraction uses CNN layers to recognize increasingly complex patterns and objects. CNNs can classify images by generating a probability distribution over output categories [75]; for example, CNNs can recognize e-cigarettes and other similar items in images. After pretraining, the CNN classification layers (which classify images) are removed, leaving only the feature extraction layers [76]. These feature extraction layers extract high-level attributes from images, converting input images into feature vectors [77]. These feature vectors are useful for image clustering because they capture image shapes, textures, and object parts.

We used the *PyTorch* library [78] for the image clustering. We resized the images to a uniform size of 224×224 pixels. We converted the images to a tensor format, which is a multidimensional array or data structure that generalizes scalars, vectors, and matrices, suitable for deep learning. We used the *DataLoader* function in *PyTorch*, which creates batches of images from the data set to be used during model training and evaluation from the data set. The function shuffles the data to ensure randomness during training. Next, we loaded a pretrained CNN model (ie, Visual Geometry Group with 16 layers [VGG16] and ResNet with 50 layers [ResNet50]), which is a built-in deep neural network model that has been trained on a large data set for image classification tasks. We specifically opted for the VGG16 [79] and ResNet50 [80] models due to their widely recognized reputation for robustness and accuracy in tasks related to image recognition [81,82]. We removed the final fully connected layers from the model, leaving only the convolutional layers. These convolutional layers were used for feature extraction. The images were passed through the VGG16 network, and the output features were collected. These features capture high-level visual information about each image and were used for clustering.

Features extracted from the images were stored in the *features* list. We turned on the evaluation mode of the model and then iterated a loop through the batches of images in our data set. For each batch, the model processes images without gradient computation (no gradients are backpropagated) using *torch.no_grad()*, and the output features are appended to the features list. The extracted features were concatenated into a single tensor and reshaped for subsequent clustering. We then determined the optimal number of clusters for the features we obtained using the silhouette score. The silhouette score measures how similar each data point in 1 cluster is to other data points in the same cluster compared with data points in other clusters. Our process involved iterating through different numbers of clusters, ranging from 2 to 10, to explore various clustering configurations. For each iteration, we computed the silhouette score for the corresponding clustering number. The silhouette score ranges from –1 to 1, where a higher score indicates better-defined clusters.

By systematically evaluating the silhouette scores across different cluster numbers, we identified the number of clusters associated with the highest silhouette score, indicating the optimal clustering number. After determining the optimal number of clusters, we performed k-means clustering using this number of clusters. The k-means algorithm groups the image features into clusters based on their similarity. We later saved images belonging to each cluster in separate subdirectories. For each cluster, we also created a CSV file to store information about the images in this cluster for qualitative analysis.

### ViT-Based Clustering

The ViT extends the transformer architecture to process images for computer vision by treating them as nonoverlapping patches or tiles, in contrast to CNNs, which process images as pixels. The ViT model treats each patch as a “word,” using self-attention mechanisms such as natural language processing transformers [83]. This allows the model to capture image patch dependencies and relationships over time. In the ViT, positional embeddings aid the model in understanding the relative positions of patches in an image [84]. Maurício et al [85] discovered that ViTs perform similarly to traditional CNNs in image classification benchmarks. The ViT model was used in recent health studies and performed well [86,87]. The feature extraction process for ViTs is similar to that for CNN models. We extracted features from our data set images using pretrained ViT models (eg, ViT-base and ViT-large). When trained on large image data sets, these models can encode images into rich feature vectors. The ViT model is used to extract features from images. The model transforms each image into a high-dimensional feature vector (representation). This vector represents the content and appearance of the image. We used *Transformers* [88], a *PyTorch*-based Python library created by Hugging Face [89], to implement the ViT model. We specifically chose the ViT model due to its proven effectiveness as a transformer-based model in comprehending visual content through tokenization [90].

After data transformation (resizing and converting into tensors, as explained previously), we initialized the ViT model using the *ViTFeatureExtractor* and *ViTForImageClassification* functions. Due to its computing capabilities, we used the *vit-base-patch16-224-in21k* model for the pretrained model. The model’s name suggests that it is moderately sized (“base”), processes images as 16×16 pixel patches, accepts input images of size 224×224 pixels, and has been trained on a data set containing 21,000 distinct image classification categories. The code iterates through the data set to extract image features using the ViT model, and the extracted features are stored in the features list and concatenated into a single tensor for further analysis. We replicated the same step from CNN-based image clustering for the feature-based clustering task. We computed the silhouette score to determine the optimal number of clusters.

### Video Classification

We switched from clustering to classification to evaluate the quality of the clusters produced by the unsupervised k-means algorithm in each model. This approach was used to determine whether the features learned during the clustering phase are informative and can be used to predict cluster assignments accurately. We divided all features and cluster labels into training (80%) and testing (20%) data sets for classification. We used a simple neural network classification model with 2 linear layers and rectified linear unit activation in between. Rectified linear unit is an activation function commonly used in artificial neural networks, particularly in deep learning models. The model underwent training with cross-entropy loss and the Adam optimizer. Cross-entropy loss or log loss is a measure of how well a probabilistic classification model predicts the probability distribution of the true labels given the input data. The Adam optimizer efficiently adjusts the model’s parameters during training by adapting learning rates and incorporating momentum that accelerates the learning in the relevant directions [91]. We trained the model for 10 epochs on each theme. We then calculated the *F*_1_-score, which is a classification metric that combines precision and recall into a single value and provides a balanced measure of a model’s performance.

### Qualitative Thematic Analysis

After obtaining the output clusters from 3 individual models (ie, ViT, VGG16, and ResNet50), we checked the samples from each cluster for each model to verify the clustering result. We determined the best model based on the performance; next, we performed qualitative thematic analysis on each theme after studying the final clustered images for each cluster on the themes. In validating the image clusters, we encountered challenges associated with interpreting visual similarities and patterns. To overcome this hurdle, we conducted a thorough examination of the clustered images, comparing them both within and across clusters to verify the consistency and accuracy of our clustering results. We initially picked 25% of the samples from each cluster, comprehensively analyzed each cluster’s images to determine whether the images were correctly clustered, and examined the characteristics of the cluster. For the second phase, we randomly selected 50 videos from each of the 5 themes to compare the content against the theme descriptor.

## Results

### Text Clustering Results

#### Overview

**Figure 3:**
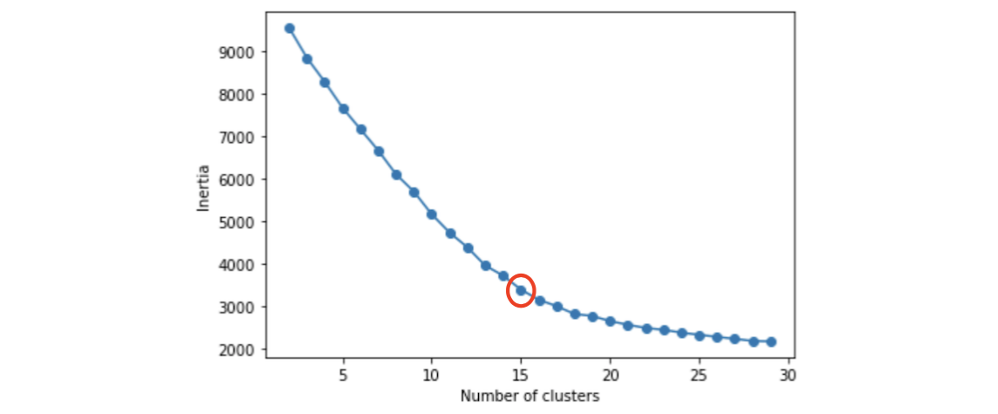
Elbow method result from k-means text clustering indicating that the optimal number of clusters is 15.

The elbow method determined the optimal number of clusters in the textual data set to be 15 (Figure 3). Our final sample was 14,002 TikTok posts after deduplication, but 3012 (21.51%) posts were unrelated to our research scope and removed from our sample, leaving 10,990 (78.49%) e-cigarette–related posts (see Multimedia Appendix 2 for details), from which we successfully identified 5 themes (summarized in Table 1).

**Figure 4:**
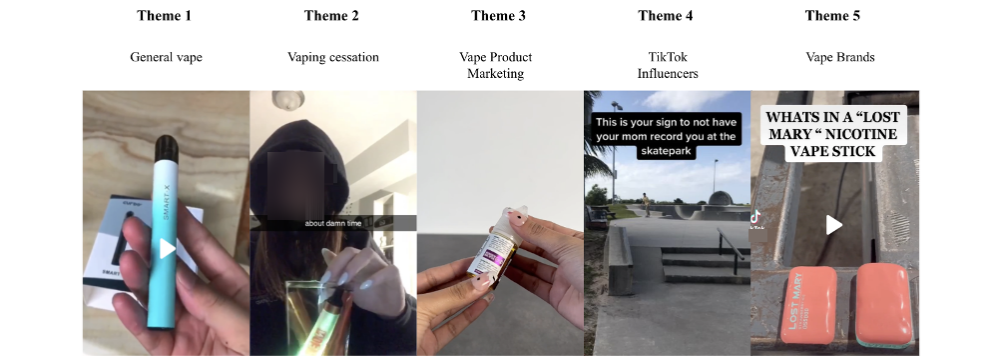
An example of each of the themes derived from text clustering (selected representative images from actual posts corresponding to each theme).

The computational textual analysis identified 5 distinct clusters, and our qualitative analysis indicated that these 5 clusters were organized by the following themes: *general vape*, *vaping cessation*, *vape product marketing*, *TikTok influencer*, and *vape brands*. These themes provided insights into the web-based discourse surrounding vaping, from general discussions to specific aspects such as cessation, influencers, and brands (Figure 4).

**Table 1:** Results of the textual thematic analysis.

Themes	Aggregated clusters (n=12), n (%)	Posts (n=10,990), n (%)	Indicators (words or hashtags)
General vape (including components, eg, coil, cartridges, and pod, as well as use, eg, blinker)	4 (33.33)	2741 (19.58)	vapetricks, cigarroeletronico, pod, vapelife, smoke, vapeo, vapor, vapemod, vapers, coil, vapepen, vapersontiktok, nicotine, know, funny, smoke, y’all, relatable, stop, vapeporn, vapefamily, wonderwaterdrip, girlvaper, vapersontiktok, carts, blinkersonlyfoo, fakecarts, blinkereyes
Vaping cessation	1 (8.33)	1272 (9.08)	vapelife, vapingislife, quit, addiction, quitvaping, health, vaping is bad, stop, stopvaping
Vape product marketing (including flavors)	1 (8.33)	1160 (8.28)	ultimatejuice, flavour, liquid, ultimatepuff, premiumliquid, eliquidshop
TikTok influencer (includes skate, gamer, vape, and other influencers)	3 (25)	3775 (26.96)	skateboarding, skater, nintendo, vapetiktoker, game, nintendo, retro, xbox, retrogrames, videogames, sega, console, games, gaming, thevideogamecollector, kingtinotazo (specific influencer)
Vape brands	3 (25)	2042 (14.58)	elf, geekbar, elfbars, pods, fresh mary, whereismymary, spookymary, scarymary, lostmarybm600 Juulgang, juullife, juulchallenge, juuling, juulsquad, mint, mango

#### Theme 1: General Vape

This theme encompassed general discussions and content regarding vaping, such as its components and use. *General vape* consisted of a substantial number of posts (2741/14,002, 19.58%) and a variety of indicators, including *vapetricks*, *coil*, and *vapelife*. These posts seemed to focus on various aspects of vaping, from the technical, such as coils and pods, to the experiential, such as user experiences. In addition, terms such as *blinker*, which is a light that flashes on and off to prevent overheating of the vaping device, suggested discussions of vaping techniques and tricks. This theme reflected a diverse and active vaping community on TikTok, engaging in discussions about their vaping experiences and preferences and possibly promoting or sharing vaping-related knowledge.

#### Theme 2: Vaping Cessation

This theme had the second lowest number of posts (1272/14,002, 9.08%). This theme discussed quitting vaping, addressing addiction, and vaping’s negative health effects. Indicators such as *quitvaping*, *stopvaping*, and *vaping is bad* suggested that a large number of individuals used social media platforms to seek support, share their experiences, and declare their intentions to quit vaping. This theme emphasized the awareness and concerns regarding the addictive nature of vaping, as well as the efforts of some vapers to overcome their addiction.

#### Theme 3: Vape Product Marketing

This theme pertained to the marketing of vape products, with a particular focus on flavors, and was characterized by a substantial post number (1160/14,002, 8.28%). The posts associated with this theme often included indicators such as *ultimatejuice*, *flavour*, *liquid*, *ultimatepuff*, *premiumliquid*, and *eliquidshop*. These indicators highlighted the prominence of discussions and content related to the marketing and promotion of various vape products, with an emphasis on the diverse range of flavors available in the vaping industry. This theme reflected the significance of flavor offerings in the marketing strategies of vape product manufacturers and highlighted the considerable attention and discourse surrounding this aspect within the vaping community or industry.

#### Theme 4: TikTok Influencer

This theme comprised a significant portion of the conversation (3775/14,002, 26.96%), entailing discussions regarding TikTok influencers and focusing on skateboarding, gaming, and vaping categories. The use of indicators such as *skateboarding*, *gamer*, and *vapetiktoker* emphasized the diversity of the discussed influencers. This theme indicated that influencers may have shared their content or discussed their impact on the platform. This theme reflected the prominent role of influencers in shaping web-based conversations, especially on platforms such as TikTok.

#### Theme 5: Vape Brands

This theme focused on specific vape brands and their products, accounting for 14.58% (2042/14,002) of the posts. The *vape brands* theme included terms such as *Juulgang*, *elfbars*, and *mint*. Users engaged in conversations regarding various e-cigarette brands, potentially discussing product preferences, flavors, and experiences. Specific brand names such as *Juul* indicated that many users were discussing this brand. This theme focused on the impact of brand loyalty and product diversity on the vaping community.

### Video Clustering Results

#### Overview

We chose to use the ResNet50 model for the qualitative thematic analysis based on the *F*_1_-score. Table 2 displays the ResNet50 model’s consistently high performance on each theme (average *F*_1_-score: 0.97), which is likely due to the improved performance of neural networks with more layers.

The image clustering results for each theme from the preceding textual thematic analysis revealed the normalization of vaping on TikTok; for example, TikTok influencers in domains such as skateboarding and gaming and TikTok vloggers uploading their daily shopping lists depict vaping as an ordinary component of daily life. Furthermore, the individuals in these videos are shown vaping in various locations (eg, at home or in a car). These findings suggest a concerning trend of normalizing vaping as an everyday practice on TikTok.

**Table 2:** Performance metrics for video classification for each model. The residual network with 50 layers (ResNet50) model performed consistently well across all 5 themes compared with the other 2 models.

	*F*_1_-scores				
	General vape (theme 1)	Vaping cessation (theme 2)	Vape product marketing (theme 3)	TikTok influencer (theme 4)	Vape brands (theme 5)
ResNet50	0.974	0.972	0.960	0.975	0.975
VGG16^a^	0.932	0.986	0.953	0.955	0.980
ViT^b^	0.943	0.941	0.847	0.986	0.778

^a^VGG16: Visual Geometry Group model with 16 layers.

^b^ViT: vision transformer.

#### Theme 1: General Vape

**Figure 5:**
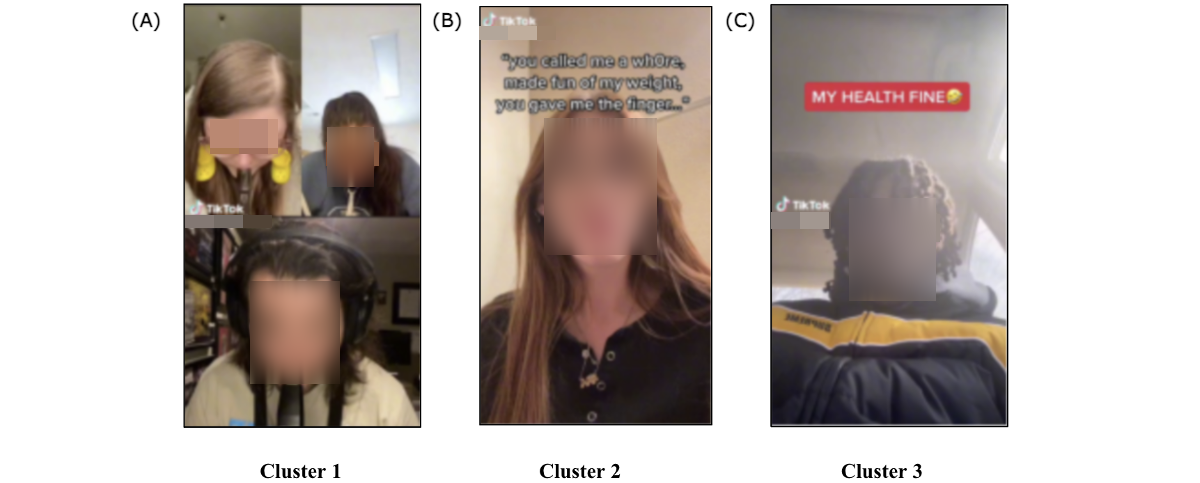
Sample image clustering results from theme 1 (general vape); 3 distinct subclusters were formed. (A) Videos portraying individuals actively participating in the act of vaping by using vaping devices. (B) Videos featuring individuals who choose not to use vaping devices, opting instead to partake in conversations that are unrelated to the act of vaping. (C) Videos showcasing individuals participating in conversations that cover a range of topics, including vaping-related subjects such as the demonstration of vaping devices as well as broader subjects such as personal health in which the creators seek to satirize antivaping messages.

The findings of our image clustering analysis indicated that the images in the *general vape* theme formed 3 distinct subclusters. We characterized each subcluster individually after carefully examining the images (Figures 5A-5C).

#### Theme 2: Vape Cessation

**Figure 6:**
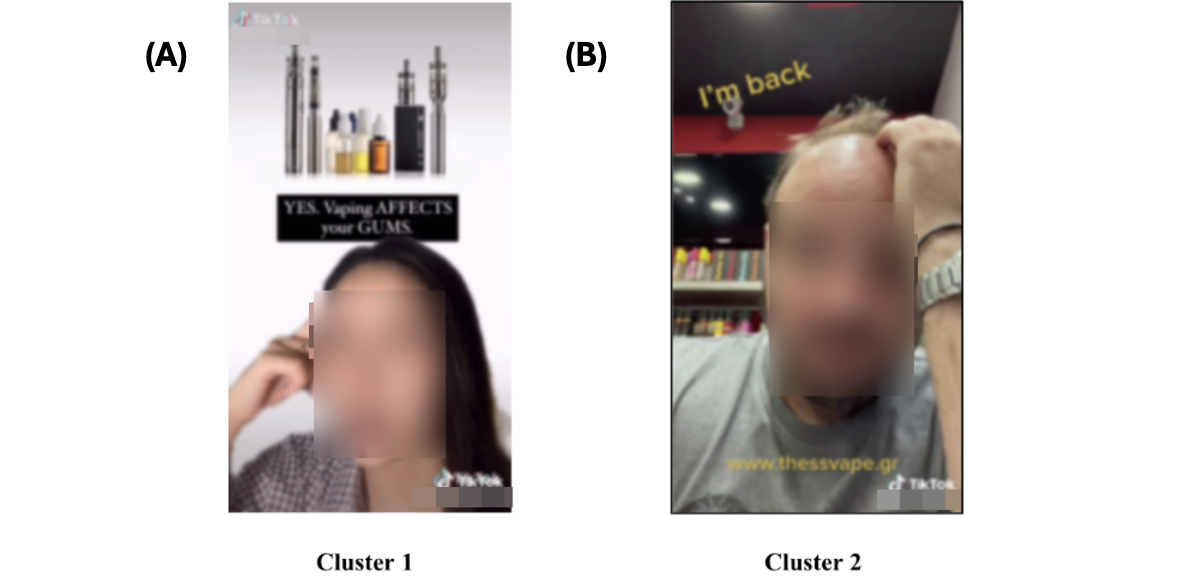
Sample image clustering results from theme 2 (vape cessation); 2 subclusters were formed. (A) Experts or users with knowledge about vaping come on screen to inform video viewers about the link between vaping and potential diseases (eg, lung-related diseases) and urge them to quit vaping. (B) Users who are actually quitting vaping or have quit vaping share their own experiences.

The findings of our image clustering analysis indicated that the images in the *vape cessation* theme formed 2 distinct subclusters: (1) experts or users with knowledge about vaping come on screen to inform video viewers about the link between vaping and potential diseases (eg, lung-related diseases) and urge them to quit vaping (Figure 6A), and (2) users who are actually quitting vaping or have quit vaping share their own experiences (Figure 6B).

#### Theme 3: Vape Product Marketing

**Figure 7:**
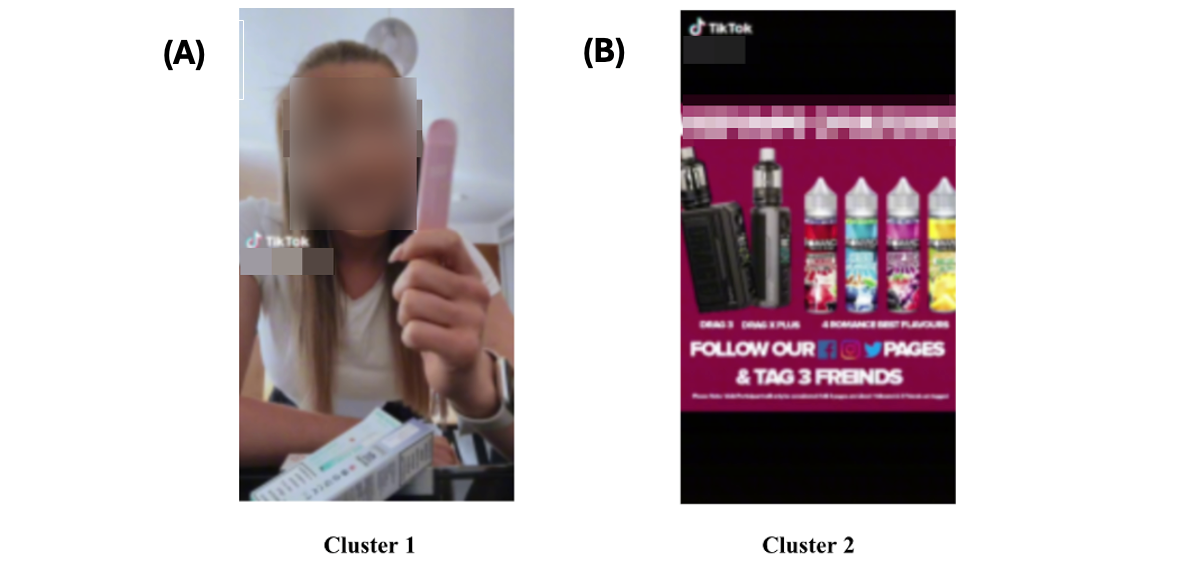
Sample image clustering results from theme 3 (vape product marketing); 2 subclusters were formed. (A) Videos that actively showcased and introduced vaping devices, illustrating the various aspects and functionalities associated with these products. (B) Videos that featured local shops actively selling and advertising vaping devices, indicating a localized and direct promotional approach.

We identified 2 distinct subclusters within the *vape product marketing* theme: (1) videos that actively showcased and introduced vaping devices, illustrating the various aspects and functionalities associated with these products (Figure 7A); and (2) videos that featured local shops actively selling and advertising vaping devices, indicating a localized and direct promotional approach (Figure 7B). Despite TikTok’s explicit guidelines opposing the direct promotion of vaping products, these subclustering results underscore the effectiveness of direct advertising or marketing of vaping devices at the microlevel, encompassing both individual users and local shops. This observation sheds light on the nuanced ways in which TikTok’s platform is used for promotional activities that may, at times, circumvent platform guidelines to reach a targeted audience.

#### Theme 4: TikTok Influencer

**Figure 8:**
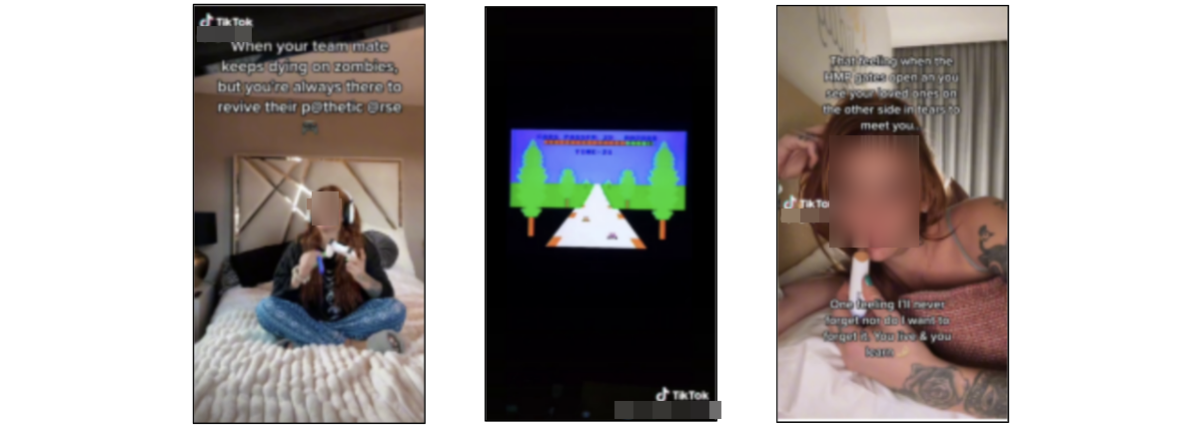
Sample image clustering results from theme 4 (TikTok influencer); no differences were found between the images within each subcluster.

We could not find differences between the images in each subcluster within the *TikTok influencer* theme. These findings demonstrate that the output of a black box computer algorithm cannot be wholly trusted. Instead, a human-in-the-loop process, in which researchers interpret the results and determine their significance, is required. Although a vaping device was visible in the video, it was not the focal point but a supplementary element. In other words, TikTok videos in the *TikTok influencer* cluster were depicting vaping alongside daily or quotidian activities such as playing video games, driving, grocery shopping, and reading books (Figure 8). There were also vape influencers in the videos, but instead of actively promoting vape products, they flaunted adding new decorations to vapes or customizing cartridges. In other words, most images within the *TikTok influencer* theme depicted vaping as a natural part of daily life.

#### Theme 5: Vape Brands

**Figure 9:**
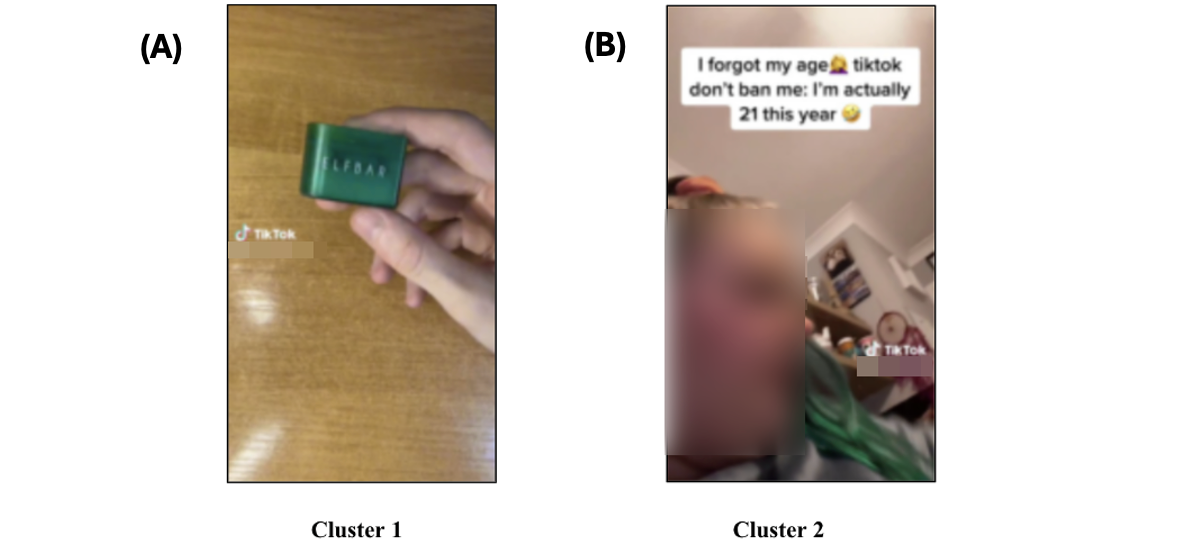
Sample image clustering results from theme 5 (vape brands); 2 subclusters were formed. (A) Videos demonstrating how to use a vaping device after opening the packaging, reviewing the product, and vaping. (2) Vaping-related context and vape use in daily settings. A TikTok user uploaded a shopping haul–related video, in which she displays a new vape (Lost Mary) and declares, “TikTok, please don’t ban me; I’m actually 21 this year.”.

We identified 2 distinct subclusters within the vape brands theme (Figures 9A and 9B). In other videos, point of view (POV) is frequently used as a text caption. POV is frequently used in captions and on-video text captions to indicate that the viewer should view the scene from their (the creator’s) POV; for instance, a user uploaded a video with the text caption “POV: you discovered elf bar made a calendar” and sang along with the background music, which contains the lyrics “Oh, I want you so badly. It’s my greatest wish.” These findings demonstrate that TikTok users are actively participating in normalizing vaping as an everyday practice. This is exemplified by the platform’s how-to videos and everyday vaping scenarios, which reflect a concerning trend in the platform’s content.

### Qualitative Thematic Analysis Results

In our qualitative thematic analysis, 50 videos were randomly selected from each of the 5 themes. These videos were viewed, and the theme of individual videos was compared with the theme descriptor to validate the themes.

Of the 50 videos selected at random from theme 1 (*general vape*), 40 (80%) were vape related; the 10 (20%) videos that were not vape related included a concert in Kuala Lumpur, Malaysia (which featured a smoky environment; n=1, 10%); a public service announcement vehicle (which featured a blue blinking light; n=1, 10%); car-related videos featuring blinkers (n=3, 30%); a festive scrunchy (n=1, 10%); a disposable camera (n=2, 20%); an advertisement for JLCPCB, a Chinese company known for manufacturing printed circuit boards (blinking circuits; n=1, 10%), and a calligraphy pen (n=1, 10%).

For theme 2 (*vaping cessation*), of the 50 videos selected at random, only 2 (4%) were not vape related (video promoting clothing: n=1, 50%; video promoting instant coffee: n=1, 50%). Of the 48 vape-related videos, 15 (31%) covered content directly related to vaping cessation, and 2 (4%) were related to quitting cigarettes (video suggesting that vaping was the best way to quit cigarettes: n=1, 50%). Of the 15 videos promoting cessation, 3 (30%) used dentists; several videos (4/15, 27%) provided tips on how to quit vaping; and others (8/15, 53%) shared information on the health and mental health benefits of quitting, such as feeling good after quitting vaping.

Of the 50 videos selected at random from theme 3 (*vape product marketing*), 13 (26%) were not vape related. The TikTok videos that were not vape related included an episode of a story (1/13, 8%), live music concerts that were filled with smoke (3/13, 23%), a girl bemoaning the loss of her boyfriend (1/13, 8%), discussions of video games (4/13, 31%), World War I Prussian rifle cartridges (1/13, 8%), a Wing Stop taste tester (who mentions a “hot box” meal; 1/13, 8%), a tattoo pen (1/13, 8%), and a calligraphy pen (1/13, 8%).

Only 1 (2%) of the 50 videos selected at random from theme 4 (*TikTok influencer*) was not vape related. Of the 49 vape-related videos, 6 (12%) conveyed an antivaping message, and 1 (2%) showed the aftermath of a vaping device exploding in a bedroom. The majority of the videos (42/49, 86%) shared images of vape pens, vape juice, and people in vape shops and presented positive images of young people vaping.

Finally, of the 50 videos selected from theme 5 (*vape brands*), 7 (14%) were not vape related. These included a video by “coolkidclaire” (1/7, 14%), boys dancing or playing football (which could have been mistaken for blinking; 3/7, 43%), a clothing store (1/7, 14%), food (1/7, 14%), and a car (another possible blinker-related error; 1/7, 14%).

Of the 250 videos reviewed, 33 (13.2%) were not vape related. However, most of these non–vape-related videos (8/33, 24%) featured a smoke-filled environment, included blinking in some form, or displayed the image of a pen. As such, most of the videos (13/33, 39%) included words or aspects of images that were similar in nature to vaping-related content and content that could easily be misclassified by a machine, highlighting the need for human interaction in this process. Moreover, only theme 2 included a variety of vaping-related images that were not related to the theme’s descriptor of *vaping cessation*. Although theme 4 (*TikTok influencer*) included some videos (7/50, 14%) providing tips on how to quit vaping and encouraging quitting, only 15 (30%) of the 50 videos from theme 2 (*vaping cessation*) conveyed this message. Again, in comparison with the other 4 themes in which most videos fit the general descriptor of the theme, this was less the case with TikTok videos related to quitting vaping. Thus, the only theme in which there was any noteworthy misclassification was in theme 2, *vaping cessation*, wherein only 15 (30%) of the 50 videos were specifically cessation related.

However, misclassification was not a failure of the model because (1) only 2 (4%) of the 50 videos in theme 2 were not directly vape related; and (2) classifying cessation-related content was challenging due to the complexity of the material, as well as the use of humor and double entendres by TikTok video creators. Many TikTok videos were characterized by a light and fun tone through the juxtaposition of ideas, words, and images, a style that was common in many of these vaping-related videos (149/250, 59.6%). Moreover, although relatively few in number (48/250, 19.2%), these vaping cessation videos are important because of the tone used by the creator, a tone that might resonate well with other TikTok users.

Finally, the nature of the misclassifications with theme 2 underscores the possibility that when humor and double entendres are an integral aspect of the message, computer-driven classification will benefit from maintaining a human-in-the-loop approach. While we can rely on machine learning algorithms to sort through thousands of images and text entries, improving the accuracy of the process requires humans to interpret images and text that are context specific and rely on socially inferred meaning.

## Discussion

### Principal Findings

This study is one of the first to use a combination of computational methods to analyze both text and image data from a large set of vaping-related content posted on TikTok between 2018 and 2023. Our analysis of the posts (n=10,990) identified 5 main themes: *general vape*, *vaping cessation*, *vape product marketing*, *TikTok influencer*, and *vape brands*. Notably, the majority of these themes (3/5, 60%) indirectly promoted vaping and may contribute to creating a web-based environment that normalizes e-cigarette use. Only one theme themes (1/5, 20%) conveyed antivaping messages; the theme identified as *vaping cessation* included more posts that indirectly promoted vaping and contributed to creating a web-based environment that normalizes e-cigarette use rather than supporting vaping cessation.

We found a relatively low proportion of posts (1160/10,990, 10.56%) explicitly engaged in direct marketing. It is noteworthy that when direct marketing was observed, it primarily focused on promoting various vaping brands and flavors. Our findings of the indirect promotion of vaping products on TikTok, particularly the promotion of vaping brands and flavors, align with those of the study by Vassey et al [45], which revealed a significant increase in the prevalence of e-juice flavor names and e-cigarette brand names on Instagram and TikTok. Of note, existing studies indicate that the variety of available flavors, ranging from fruity to sweet and other appealing tastes, is one of the top reasons cited by young people to experiment with e-cigarettes [92,93].

Further findings indicated that popular TikTok users or influencers primarily promoted vaping products indirectly. Influencers, often associated with youthfulness on social media, are skilled at subtly endorsing products, thereby making them more relatable than traditional advertisements. The findings showed that influencers make vaping look like a natural part of their daily lives as they engage in activities such as gaming, partying, traveling, and so on, which may lead to indirectly normalizing the behavior. Brands partnering with influencers can effectively shape attitudes and behaviors because it does not trigger the same resistance as overt direct advertising [94]. Nonetheless, these findings align with and expand prior research documenting a notable presence of influencers’ role in promoting vaping on social media platforms on behalf of tobacco brands [1]; for example, a recent study showed that 20% of the influencers exclusively posted about e-cigarette use on Instagram, while 80% of them posted about e-cigarettes and other topics [3]. Moreover, when influencers create content related to vaping, TikTok algorithms actively promote this content to a larger audience, significantly expanding its reach and impact [94].

Our study identified a substantial amount of content that violates TikTok’s current policies, underscoring the need for stricter regulations in web-based advertising, particularly when the main companies use indirect methods through smaller sellers or influencers to promote their vaping products. Furthermore, the normalization of vaping through influencers or other strategies poses a significant concern for public health, especially among younger people who are more susceptible to the influences of such advertising [95].

### Implications for Policy and Research

Given the findings of this study, social media platforms should consider implementing more robust measures to ensure that videos reach only their intended audiences; for example, social media platforms should (1) require age verification for users accessing any vaping content or engaging with vaping-related content, (2) require enhanced posts that highlight the potential health risks associated with vaping, and (3) prevent content that contains misleading information about the safety of vaping. Policy makers could develop regulations to enforce stricter advertising policies, explicitly prohibiting the promotion of vaping products and restricting content that indirectly promotes vaping [96,97].

Policy makers may also consider implementing advanced algorithms to detect and flag content that indirectly promotes vaping. These algorithms can identify visual patterns and keyword text associated with vaping-related content. Furthermore, policies should develop and enforce specific guidelines for influencers, including transparency requirements regarding affiliations with vaping brands and restrictions on promoting vaping to underage populations. In addition, investing in the development of prevention programs aimed at denormalizing vaping use among youth could be crucial in mitigating the spread of vaping culture and its associated risks.

### Future Work and Limitations

We successfully implemented a novel approach that combines 2 machine learning methods—natural language processing and computer vision—alongside qualitative thematic analysis. Specifically, our approach leveraged computational methods, specifically k-means clustering for textual data and state-of-the-art ViT and CNN models for image data. A computational, machine learning–based methodology allowed us to analyze a large volume of textual and visual content efficiently. We uncovered key insights into the prevalence and acceptance of vaping on TikTok using these computational techniques. However, we acknowledge a few limitations. Incorporating video transcription software, which automatically transcribes spoken language into written text, could provide additional textual data for analysis in future work. This enhancement could significantly improve the text corpus for thematic analysis. In addition, due to our hashtag-based data scraping approach, we encountered limitations in extracting comprehensive vaping-related information; for instance, our objective was to scrape content from posts containing the hashtag *#blinker*, which denotes a visual indicator akin to the light-emitting diode lights found on a vaping device, including an e-cigarette or a vape pen. However, our scraped results included irrelevant content, such as a public service announcement vehicle with a blue blinking light and a calligraphy pen. The algorithmic flow of TikTok necessitates a human-in-the-loop approach, which we implemented successfully in this study.

Future work could benefit from incorporating video transcription software, further enriching textual data for even more comprehensive thematic analysis. Although our classification of vaping-related messages was successful (average *F*_1_-score=0.97), our classification of vaping cessation messages presents opportunities for further improvement. Specifically, cessation-related messages are difficult for machines to understand because they contain subtle cues (eg, humor). These findings indicate that humans are vital to the classification process. Future work should focus on developing methods to enhance a machine’s ability to interpret context-specific images and text and extract socially inferred meaning in complex areas such as humor and vaping cessation. Furthermore, exploring the integration of more advanced natural language processing models, such as Bidirectional Encoder Representations from Transformers [98], could further enhance the depth and accuracy of our text analysis. These models offer contextual understanding, capturing intricate relationships within text that may provide deeper insights into the themes and sentiments expressed in vaping-related content on TikTok.

### Conclusions

This study conducted a thematic analysis of vaping-related content on TikTok, using a hybrid computational and qualitative approach to analyze textual and visual data, respectively, to ensure precision and reduce bias. We aimed to discern the patterns and themes within vaping-related content to understand the extent to which vaping is normalized in the context of social media discourse. We conducted a multifaceted investigation into user-generated content on social media platforms related to e-cigarette (vape) discussions, focusing specifically on TikTok. We found that vaping-related content on TikTok is marked by distinct themes, including those portraying vaping as a common activity in daily life, discussions on vaping cessation, TikTok influencers incorporating vaping into their content, and specific discussions related to vape brands and product marketing. These themes provided valuable insights into the normalization of vaping on the platform. Images within each clustered theme highlighted the various facets of vaping, such as active vaping practices, conversations unrelated to vaping but with a vaping device visible in the video, and vaping as an integral part of daily life.

Consistent with these findings, the qualitative analysis revealed that our machine learning approach correctly recognized vape-related content by successfully parsing these data into smaller meaningful units (ie, the 5 themes). The cessation-related TikTok videos merit additional comment and research. They are few in number (1272/14,002, 9.08%), but this is unsurprising because they are created by TikTok users. However, their impact may be far greater than officially created content because the user-generated approach of cessation messaging might be far more appealing to other TikTok users than cessation messages created by professionals.

Overall, our computational methods with a qualitative human-in-the-loop approach allowed us to gain a deeper understanding of how vaping is represented and normalized on TikTok. Our study provided a broader perspective, emphasizing the normalization of vaping practices rather than focusing solely on the textual or visual content of prior research. Moving forward, our findings underscore the importance of ongoing surveillance of vaping-related content on social media platforms, highlighting the need for targeted interventions to mitigate the normalization of harmful behaviors among youth populations.

## Appendix

Multimedia Appendix 1 List of TikTok hashtags. We scraped the data using 50 hashtags.

Multimedia Appendix 2 Text clustering result. We identified 15 clusters using the k-means clustering method.

## Abbreviations

API: application programming interface
CNN: convolutional neural network
POV: point of view
ResNet50: residual network
TF-IDF: term frequency–inverse document frequency
VGG16: Visual Geometry Group with 16 layers
ViT: vision transformer

## Data Availability

The data sets and code generated and analyzed during this study are available from the corresponding author on reasonable request.
