# Supplementary material for: The Normalization of Vaping on TikTok Using Computer Vision, Natural Language Processing, and Qualitative Thematic Analysis: Mixed Methods Study
Source: J Med Internet Res. 2024 Sep 11;26:e55591. doi: 10.2196/55591 (PMC11425021; doi:10.2196/55591)
Supplement: Multimedia Appendix 1 [file jmir_v26i1e55591_app1.docx]

**Multimedia Appendix**

**Appendix** 1: List of TikTok hashtags. We scraped the data with 50 hashtags.

| **Selected Hashtags**  #boxmod  #boxmods  #disposablevapepen  #disposablevapor  #ejuice  #ejuiceflavor  #disposablecarts  #disposablecart  #disposables  #disposable  #vapetok  #vapetik  #cartridge  #eliquid  #eliquids  #eliquidflavors  #vapejuice  #juul  #vape  #juulpods  #juuls  #juulpod  #vaping  #vapingcommunity  #vapinggirls  #hotbox  #vapear  #freshvapes  #vaper  #vapesession  #blinker  #blinkers  #blinkerchallenge  #geekvapetech |
| --- |
