# Supplementary material for: The Normalization of Vaping on TikTok Using Computer Vision, Natural Language Processing, and Qualitative Thematic Analysis: Mixed Methods Study
Source: J Med Internet Res. 2024 Sep 11;26:e55591. doi: 10.2196/55591 (PMC11425021; doi:10.2196/55591)
Supplement: Multimedia Appendix 2 [file jmir_v26i1e55591_app2.docx]

**Multimedia Appendix**

**Appendix** 2: Text clustering result. We identified 15 clusters from K-Means clustering method.

| Cluster Num | Theme | Indicator (word or hashtag) |
| --- | --- | --- |
| Cluster 0 | General vape (includes tricks, nicotine discussion, coils) | Vapetricks, cigarroeletronico, pod, vapelife, smoke, vapeo, vapor, vapemod, vapers, coil, vapepen, vapersontiktok, nicotine |
| Cluster 1 | Vaping cessation | Vapelife, vapingislife, quit,  Addiction, quitvaping, health, vaping is bad, stop, stopvaping |
| Cluster 2 | Vape product marketing | ultimatejuice, flavour, liquid, ultimatepuff, premiumliquid, eliquidshop |
| Cluster 3 | TikTok InfluencMultimedia Appendix  er | Skateboarding, skater, nintendo, vapetiktoker |
| Cluster 4 | TikTok Influencer | Game, nintendo, retro, xbox, retrogrames, videogames, sega, console, games, gaming, thevideogamecollector |
| Cluster 5 | TikTok Influencer | Teamteemo, kingtinotazo, tinotime |
| Cluster 6 | General vape (Cartridges, Blinker) | Carts, blinkersonlyfoo, fakecarts, blinkereyes |
| Cluster 7 | Unrelated (e.g.,The Musical Wicked) | Elphabaorion, elphabadoherty, elphabalive, defyinggravity |
| Cluster 8 | Vape Brands (Elfbar-related) | Elf, geekbar, elfbars, pods |
| Cluster 9 | Spanish-language | Vapeo, que, venezuela, vapeador, cigarrilloelectronico, desechable, vaporesso,españa,dejardefumar,vapeadores,vapeocolombia,vapearcolombia |
| Cluster 10 | Unrelated (e.g, Disposable Camera) | Disposablecamera, film, review, papershootcamera, papershoot, photography |
| Cluster 11 | General vape | Vapeporn, vapefamily, wonderwaterdrip, girlvaper, vapersontiktok |
| Cluster 12 | General vape | Nicotine, know, funny, smoke, y’all, relatable, stop |
| Cluster 13 | Vape Brands (lostmary-related) | fresh mary, whereismymary, spookymary, scarymary, lostmarybm600 |
| Cluster 14 | Vape Brands (Juul-related) | Juulgang, juullife, juulchallenge, juuling, juulsquad, mint, mango |
